# Current Treatment of Heart Failure with Preserved Ejection Fraction: Should We Add Life to the Remaining Years or Add Years to the Remaining Life?

**DOI:** 10.1155/2013/130724

**Published:** 2013-10-24

**Authors:** Jia Li, Peter Moritz Becher, Stefan Blankenberg, Dirk Westermann

**Affiliations:** Department of General and Interventional Cardiology, University Heart Center Hamburg Eppendorf, Martinistraße 52, 20246 Hamburg, Germany

## Abstract

According to the ejection fraction, patients with heart failure may be divided into two different groups: heart failure with preserved or reduced ejection fraction. In recent years, accumulating studies showed that increased mortality and morbidity rates of these two groups are nearly equal. More importantly, despite decline in mortality after treatment in regard to current guideline in patients with heart failure with reduced ejection fraction, there are still no trials resulting in improved outcome in patients with heart failure with preserved ejection fraction so far. Thus, novel pathophysiological mechanisms are under development, and other new viewpoints, such as multiple comorbidities resulting in increased non-cardiac deaths in patients with heart failure and preserved ejection fraction, were presented recently. In this review, we will focus on the tested as well as the promising therapeutic options that are currently studied in patients with heart failure with preserved ejection fraction, along with a brief discussion of pathophysiological mechanisms and diagnostic options that are helpful to increase our understanding of novel therapeutic strategies.

## 1. Introduction

Heart failure (HF) with preserved ejection fraction (HFPEF) has been well recognized as an increasing epidemiological and medical challenge over the last two decades [1, 2]. Studies indicate that the number of patients with HFPEF is similar or even higher compared to the number of patients with HF with reduced ejection fraction (HFREF) [3, 4]. Moreover, we do know that the mortality is similar in patients with HFPEF compared to HFREF [5]. However, the conventional medical therapies in HFREF, that are based on the strong evidence of multiple randomized controlled clinical trials (RCTs) showing a decline in mortality, have shown no favourable result in HFPEF so far [6, 7]. A recent study also showed that noncardiac deaths in HFPEF are higher than in HFREF, which could be a result of multiple complicating diseases in patients with HFPEF [8]. This review will focus on the tested and upcoming treatment options in HFPEF. Moreover, pathophysiological mechanisms and diagnostic options will also be briefly discussed in order to understand new therapeutic targets in this field. 

## 2. Diagnosis of HFPEF

According to the latest recommendations of the European Society of Cardiology and American Heart Association [6, 7], there are, although this is still under intensive discussion, at least three criteria for the diagnosis of HFPEF: clinical signs and/or symptoms of HF, normal or mild reduction of systolic with left ventricular (LV) ejection fraction (LVEF) > 50% with normal size of LV (LV end-diastolic volume index  < 97 mL/m^2^), and evidence of reduced diastolic LV function. This is usually determined by echocardiography (abnormalities of the mitral inflow pattern, tissue velocities (e), or the E/e ratio, left atrial volume index  > 34 mL/m^2^, and increased LV mass index) or biomarker assessment (NT-proBNP). Other cardiac aetiologies including valvular heart disease, hypertrophic cardiomyopathy, infiltrative or restrictive cardiomyopathy, and constrictive pericarditis have to be excluded carefully [9]. Nevertheless, today we do know that other causes next to diastolic dysfunction are also present and play an important role for many patients with HFPEF. These causes include, for example, endothelial dysfunction, chronotropic incompetence, impaired ventricular vascular coupling, and postcapillary pulmonary hypertension and may alter future therapeutic options [10].

## 3. Brief Introduction in the Pathophysiology of HFPEF

One leading mechanism of HFPEF is LV diastolic dysfunction. Diastolic dysfunction consists of abnormal LV active relaxation as well as increased LV passive stiffness [11]. As an energy consuming process, abnormal LV active relaxation is related to ischemia of cardiac myocytes [12] or abnormalities in myocardial energy metabolism [13]. Furthermore, increased diastolic LV stiffness limits cardiac output by elevating LV end-diastolic pressures and decreasing stroke volumes, which has been illustrated in HFPEF by invasive and noninvasive methods at rest [11, 14] or during atrial pacing and exercise [14]. The substrate of LV stiffness seems to be excessive collagen type I deposition resulting in a stiff and noncompliant extracellular matrix (ECM) [15, 16], but also titin phosphorylation deficit [16–18] is involved in this process [19–21]. Several studies from both animals and humans showed that additional mechanisms on organ level also play an important role, such as autonomic dysfunction [22], reduced vasodilator reserves [22, 23], impaired heart rate recovery and chronotropic incompetence [22, 24], diastolic and systolic dyssynchrony [25], and abnormal ventricular vascular coupling [26, 27]. Moreover, it has been proven that the renin-angiotensin-aldosterone system (RAAS) and sympathetic nervous systems were upregulated in HFPEF and hence contribute to disease progression [28, 29]. Recently, studies focused on the molecular cell level disorder in HFPEF-like impaired nitric oxide—cyclic guanosine monophosphate (cGMP)—protein kinase G (PKG) signaling [30, 31], endothelial dysfunction [22, 32, 33], oxidative stress, and cardiac inflammation [20]. The roles of comorbidities in the HFPEF population, including arterial hypertension, coronary arterial disease (CAD), diabetes, atrial fibrillation, obesity, obstructive sleep apnoea, and chronic kidney disease (CKD), are all associated with the disease and will likely be involved in the pathophysiology [34–36]. Furthermore, patients with HFPEF are older, more often female, have less often CAD but higher rates of atrial fibrillation, and most of them have arterial hypertension [37]. All of these findings may lead to a higher noncardiac death rate of nearly 30% in HFPEF and higher non-HF hospitalizations compared to HFREF [8, 38, 39]. As a result of the diversity of the underlying mechanisms and comorbidities, HFPEF presents as a complex clinical syndrome associated with multiple pathophysiological alterations rather than one single entity [40]. Taken together, this makes treating HFPEF a clinical challenge.

## 4. Pharmacological Treatment

The exact mechanisms of HFPEF are still under investigation. Nevertheless, there are several clinical trials in the HFPEF population targeting on clinical symptoms, exercise capacity, diastolic dysfunction, and quality of life (QoL). While there are tested treatments improving these outcomes, no confirmed positive outcomes in regard to mortality were obtained from all pharmacological therapies including diuretics, beta-blockers, RAAS antagonists, digitalis, HMG-CoA-reductase inhibitors (statins), nondihydropyridine calcium channel blockers, and phosphodiesterase-5 inhibition (PDE-5 inhibition) so far.

### 4.1. Diuretics

Up to now, the data about the effect on long-term prognosis of diuretics in HFPEF are still limited. In the Hong Kong Diastolic Heart Failure Study [41], diuretics (furosemide or thiazide) alone or combined with ramipril or irbesartan were evaluated in 150 patients with LVEF > 45%. Diuretic use alone was associated with improvement of symptoms and QoL significantly, while the addition of ramipril or irbesartan provided only slight additional beneficial effects. There was no survival benefit or reduction in HF hospitalization in this rather small study. Importantly, in one ALLHAT substudy, chlorthalidone reduced the incidence of new-onset hospitalization in patients with HFPEF significantly compared to patients treated with amlodipine and doxazosin. The occurrence of new-onset HFPEF was also smaller compared to lisinopril [42]. Similar results were demonstrated by HYVET (Hypertension in the Very Elderly Trial); indapamide combined with or without perindopril showed a significant HF reduction by 64% in patients of 80 years of age or older, which suggests that many suffered indeed from HFPEF. With the lack of evidence in mortality reduction, according to current guidelines [6, 7], diuretics should be used only for relief of symptoms (breathlessness and edema) due to sodium and water retention in patients with HFPEF. Nevertheless, diuretics are still an important cornerstone in daily clinical routine.

### 4.2. Renin-Angiotensin System (RAS) Antagonists

It is well known that three large RCTs have failed to reduce morbidity and mortality in patients with HFPEF. The Candesartan in Heart Failure: Assessment of Reduction in Mortality and Morbidity (CHARM)-Preserved trial [43], which showed only moderate reduction in HF hospitalization, when assessing the reduction of mortality and morbidity, showed a 30% reduction in the risk of death at 1 year (*P* < 0.001) but only a 9% reduction (*P* > 0.055) over the full-duration followup of 38 months in median. The Perindopril for Elderly People with Chronic Heart Failure (PEP-CHF) study [44] showed that angiotensin-converting enzyme inhibitors (ACEI) resulted in improved symptoms and exercise capacity and HF hospitalization but no reduction in long-term morbidity and mortality. This may result from insufficient power for lower enrolment and event rates than anticipated. Moreover, the Irbesartan in heart failure with preserved systolic function (I-Preserve) trial [45], which showed no reduction in the primary composite outcome of death or cardiovascular hospitalization. Recently, a propensity-matched inception cohort study showed that angiotensin receptor blocker (ARB) use obtained no improved clinical outcomes in real-world older (mean age of 80 years) HFPEF patients [46]. A meta-analysis of trials was reported regarding whether pharmacological agents improved exercise capacity, LV diastolic function, and survival benefit in patients with HFPEF [47]. From 18 RCTs and 12 observational studies, data of 53,878 patients were analysed. After 18.6-month followup, all-cause mortality was unimproved both in RCTs and observational studies. However, there were tendencies in these studies toward marginal benefits in primary outcomes. Considering these neutral results may be due to a bias of selection or underpowered data or high crossover rates which concealed a real benefit [48]. Recently, additional two large rigorous registry studies gave us a glimpse of the hope. An observational analysis conducted by Lund and colleagues with the Swedish Heart Failure registry showed that RAS antagonists may reduce all-cause mortality in patients with HFPEF [49]. In this trial, 16,216 patients with HFPEF (LVEF ≥ 40%, mean age 75 years; 46% women) were assessed in an age-matched and propensity score-matched cohort balanced on 43 variables. 12,543 patients treated with RAS antagonists (ACEI or ARB) and 3,673 without RAS antagonists as control group. In the matched HFPEF cohort, the treated patients versus those untreated in 1-year survival was 77% (95% CI, 75%–78%) versus 72% (95% CI, 70%–73%), respectively, (hazard ratio (HR) 0.91, 95% CI, 0.85–0.98; *P* = 0.008). In the overall HFPEF cohort, the treated patients in crude 1-year survival was 86% (95% CI, 86%-87%) versus 69% (95% CI, 68%–71%) in untreated patients (HR 0.90, 95% CI, 0.85–0.96; *P* = 0.001). In the HFPEF dose analysis, the HR was 0.85 in > 50% of target dose group versus in untreated group (95% CI, 0.78–0.83; *P* < 0.001), while HR was 0.94 in <50% of target dose group versus in untreated group (95% CI, 0.87–1.02; *P* = 0.14). It is notable in this study the LVEF ≥ 40% for HFPEF diagnosis may include the subgroup of 40–50% which represents systolic dysfunction [50]. This lax enrolment might increase more benefits of RAS antagonists than in those with LVEF ≥ 50%, as mentioned by the author that the subgroup of LVEF 40–50% have a tendency of more reduction in mortality compared to those LVEF > 50% [49]. Another similar study evaluated ACEI therapy in > 65 years older patients with HFPEF [51]. Date of patients from the Organized Program to Initiate Lifesaving Treatment in Hospitalized Patients With Heart Failure (OPTIMIZE-HF) and link to Medicare, 1,337 eligible patients (mean LVEF = 55%, mean age of 81 years, 64% women) receiving ACEI were matched by propensity scores to 1,337 patients not receiving ACEI. Initiation of ACEI was linked with a 9% modest reduction in all-cause mortality or HF hospitalization during 2.4 years of followup in median (HR 0.91, 95% CI, 0.84–0.99; *P* = 0.028). However, there is also limitation in observational studies that the decision to use the agents depends on patient factors instead of using the agents in random [52]. Recently, LCZ696, the combination of ARB (valsartan) and neprilysin inhibitors (AHU377), acts as a promising drug in hypertension and HF [53–55]. Neprilysin is able to attenuate biological active of brain natriuretic peptide (BNP), and atrial natriuretic peptide (ANP), LCZ696, increasing natriuretic peptides by inhibiting this enzyme, might result in favourable cardiovascular effects [55]. LCZ696 now is evaluated in a phase II study (The PARAMOUNT study) in patients with HFPEF [56]. All 301 patients based on a clinical diagnosis of HFPEF (LVEF ≥ 45%), with increased plasma concentration of N-terminal prohormone brain natriuretic peptide (NT-proBNP) > 400 pg/mL. 149 patients were randomly assigned to LCZ696 group, and 152 patients to valsartan group. Finally, 134 and 132 patients were included in LCZ696 and valsartan group, respectively, in total 36-week analysis of the primary endpoint. Compared with the valsartan group, the LCZ696 group achieved a 23% (*P* = 0.005) reduction in plasma NT-proBNP level after the first 12 weeks, while only 15% (*P* = 0.20) over the full 36-week followup. In addition, LCZ696 had beneficial effects on symptoms. All of the above, although suggested some benefits, required more appropriately powered RCTs in the future. Nonetheless, on the other hand, there are also no harmful pieces of evidence of RAS antagonists in patients with HFPEF. According to current guidelines, it is reasonable to use RAS antagonists for hypertension treatment in HFPEF [6, 7]. In other words, the application of RAS antagonists is not because they are of benefit in HFPEF, but for most patients with HFPEF have the indication for RAS antagonists related to comorbidities, such as arterial hypertension and diabetes [57].

### 4.3. Mineralocorticoid Receptor Antagonists (MRAs) or Aldosterone Receptor Antagonist (ARAs)

There is growing evidence to suggest that mineralocorticoid receptor antagonists (MRAs) are beneficial for the patients with HFREF. The RALES trial [58] initiated a wide use of MRAs in severe HFREF patients. Additionally, the EPHESUS study [59] led to the widespread use of these agents in postmyocardial infarction patients with HF. The EMPHASIS-HF study [60], thus, changed current guidelines with expanded use of MRAs to patients with mild HFREF. For patients with HFPEF, a small RAAM-PEF trial showed echocardiographic improvement of diastolic function and a decrease in serum markers of collagen turnover [61]. More recently, the Aldo-DHF trial investigated the efficacy of spironolactone in 422 patients with HFPEF (LVEF ≥ 50%) and mild symptoms. Spironolactone was demonstrated as the first MRA to show an improvement in diastolic function in patients with HFPEF, despite no effects on maximal exercise capacity improvement, symptoms relief, or QoL increase. However, because of a relative “healthy or young” study population, as well as the treatment period might be too short to provide useful data on clinical benefit, the Aldo-DHF trial was not powered to evaluate the role of spironolactone in HF hospitalizations or mortality [62]. An ongoing much larger TOPCAT study [63], which tries to answer whether spironolactone is of benefit in the reduction of cardiovascular death, aborted cardiac arrest, and hospitalization for HF compared with placebo in 3,445 patients with HFPEF (LVEF > 45%), will provide more data in this issue.

### 4.4. Beta-Blockers

Beta-blockers were thought of as keystone in the treatment of HFREF [6, 7]. However, the exact effect of beta-blockers treatment on patients with HFPEF still remains unclear. In the Swedish Doppler-echocardiographic study (SWEDIC), carvedilol showed echocardiographic improvement in diastolic function [64]. Nevertheless, exercise capacity [65] and mortality within beta-blockers-treated HFPEF patients remain unchanged in other RCTs [66–68]. Recently, the results of Japanese Diastolic Heart Failure (J-DHF) study were published [67]. In this study, 245 patients with HF and LVEF > 40% were randomly divided into carvedilol group and control group (without carvedilol). The primary endpoints of this study are composite of cardiovascular death and hospitalization for HF. During 3.2 years of followup in median, the endpoints occurred in 29 patients in the carvedilol group and in 34 patients in the control group (HR 0.90, 95% CI, 0.55–1.49; *P* = 0.6854). The results of any cardiovascular death and unplanned cardiovascular hospitalization were 38 patients in the carvedilol group and 52 in the control group (HR 0.77, 95% CI, 0.50–1.17; *P* = 0.2178), respectively. This study suggested that carvedilol could not improve prognosis of patients with HFPEF. However, the editorial of this study considered that the numbers of patients were probably too small to show significant differences, so they tried to pool the data of three similar small studies together and demonstrated that beta-blockers could reduce all-cause mortality in the HFPEF population [57]. This should be reassessed by another ongoing b-Preserve trial, which aimed to enroll 1,200 patients with HFPEF randomized to metoprolol or placebo therapy and to clarify the long-term effect of beta-blocker treatment in the HFPEF population [69].

### 4.5. Phosphodiesterase-5 Inhibition (PDE-5 Inhibition)

Sildenafil, as a typical agent of selective PDE-5 inhibition, is currently permitted for group 1 pulmonary arterial hypertension treatment. Recently, there are increased numbers of trials to evaluate the effects of sildenafil in chronic HF. Guazzi and his coworkers studied 44 patients with HFPEF (LVEF ≥ 50%) and pulmonary hypertension (pulmonary artery systolic pressure > 40 mm Hg). Compared with placebo, sildenafil significantly reduced mean pulmonary artery pressure and right atrial pressure, but also improved right ventricular function and symptoms [70]. Furthermore, they conducted another study in HF to demonstrate that sildenafil could improve LV diastolic function and cardiac geometry [71]. In contrast, the RELAX trial showed a different result [72]. This study aimed to evaluate the role of PDE-5 inhibition in improvement of clinical status and exercise capacity in diastolic HF. 216 patients with HFPEF (LVEF > 50%), associated with reduced exercise capacity and increased NT-BNP or elevated invasively measured LV filling pressures, were assigned to sildenafil group (*n* = 113) or placebo (*n* = 103). The primary endpoint of this study is evaluation in peak oxygen consumption after 24 weeks of therapy. Secondary endpoints were change in 6-minute walk distance and clinical status assessment. The result showed that sildenafil failed to achieve improvement in exercise capacity in patients with HFPEF. As far as now, studies on PDE-5 inhibition in HFPEF are mainly focused on clinical status improvement or symptom relief instead of mortality reduction. However, as pointed by Kitzman that improvements in symptom among HF patients might diverge from improvements in mortality. The best examples are using positive inotropic drugs in the treatment of HFREF, which resulted in the most effective improvement in symptom but worsened survival, while using beta-blockers, which worsen the symptom acutely, brings a significant reduction in mortality [73]. Thus, although exercise capacity is an important clinical endpoint and might be improved by PDE-5 inhibition in the HFPEF population, the efficacy of PDE-5 inhibition on survival benefit still needs to be evaluated in further large RCTs.

### 4.6. Nondihydropyridine Calcium Channel Blockers (Non-DHP CCBs)

In patients with HFPEF, the effects of nondihydropyridine calcium channel blockers (non-DHP CCBs) are still unknown. Published trials in this field were mainly focused on improvement of LV diastolic function and symptom relief with treatment of verapamil [74, 75]. A recent substudy of the ASCOT (the Anglo-Scandinavian Cardiac Outcomes Trial) trial demonstrated that patients receiving the amlodipine-perindopril regimen had better diastolic function than an atenolol-thiazide regimen [76]. However, it is unclear whether amlodipine or perindopril has the more important effect in this analysis.

### 4.7. Other Medications

Digoxin, compared with placebo in the digitalis investigation group (DIG) trial, showed neutral result in mortality among patients with HFPEF [77]. However, findings from a more recent study were at odds with the previous neutral results. Meyer and his coworkers found a statistically significant benefit including mortality or hospitalization with digoxin treated group after 2 years of followup, compared with placebo group in patients with HFPEF and in patients with HFREF. However, at the end of the analysis period (median 3.2 years), no significant difference was seen between digoxin and placebo in either the HFPEF group or HFREF group. This result probably was caused by the higher digoxin doses and crossover design [78]. Digoxin is only recommended for control of the rapid ventricular rate during atrial fibrillation in patients with HFPEF.

Statins, as anti-inflammatory agents, have been well demonstrated as a first-line therapy in CAD and hyperlipidemia. Neutral findings have been reported in the CORONA (Controlled Rosuvastatin Multinational Trial in Heart Failure) trial regarding the efficacy of statins in patients with HFREF [79]. However, Fukuta et al. reported a significant relative risk reduction in mortality in patients with HFPEF (LVEF > 50%) treated with statins and followed for 21 months [80]. Recent studies also suggested that statins therapy seems to be associated with improved survival benefit in patients with HFPEF [81–84]. This effect remains unclear because only some small observational studies were performed so far. 

The Ranolazine for the Treatment of Diastolic Heart Failure (RALI-DHF) trial, that was focused on the effect of ranolazine in patients with HFPEF, is undergoing now [85]. As an antianginal agent and late sodium channel inhibitor, ranolazine might improve LV diastolic function through inhibition of late sodium current or probably a direct effect on myofilament cross-bridge kinetics and myofilament sensitivity to calcium [86].

A recent epidemiological study showed that the decrease of vitamin D is associated with hypertension, LV hypertrophy, and diastolic dysfunction [87]. An additional study revealed vitamin D regulates renin transcription and the RAAS regulation by activating the vitamin D receptor [88]. An ongoing research in the Vitamin D CHF trial is currently under investigation for the change of plasma renin activity by administration of high-dose vitamin D in a stable chronic HF group [89].

Ivabradine, a drug that inhibits the *I*_*f*_ channel in sinus node, has been found to improve LV systolic and diastolic function in an angiotensin II-induced HF mouse [90]. Ivabradine might be a novel therapeutic concept for HFPEF through effects that improve vascular stiffness, LV contractility, and diastolic function [91]. It is currently also under investigation in patients with HFPEF [92].

Relaxin was evaluated in the treatment of acute HF patients in the RELAX-AHF study lately [93]. Relaxin might play a role in potential benefits in patients with HFPEF due to additional properties including antifibrosis, anti-inflammatory, and anti-ischemic [94]. 

The combination of hydralazine and isosorbide dinitrate (HISDN) has been recommended to reduce morbidity and mortality for African Americans with advanced HFREF [6, 7]. However, experimental research indicated that diastolic function and exercise capacity could be improved by HISDN [95]. In addition, a study of Vasodilators on Cardiac Function in Diastolic Heart Failure aims to assess the potential benefits of HISDN treatment in the HFPEF population [96].

## 5. Nonpharmacological Treatment

As evidence-based recommendation by current guidelines, regular exercise is implemented in patients with stable HFREF [6, 7]. Recently, the results of the Ex-DHF (Exercise training in Diastolic Heart Failure) pilot study illustrated that exercise capacity and QoL were improved by exercise training (ET) in patients with mild HFPEF. This finding is probably linked with reversed atrial remodeling and LV diastolic function improvement [97]. However, some recent studies suggested that the improvement of exercise capacity and QoL might come from nonheart organs. Haykowsky and his colleagues found the exercise capacity improvement may mainly be due to peripheral mechanisms (microvascular and/or skeletal muscle function improvement) in elderly stable compensated HFPEF patients [98]. Another study published by Kitzman and his colleagues demonstrated similar results; after 16 weeks of ET, the increase in peak VO_2_ was disassociated with endothelial dependent flow-mediated arterial dilation (FMD) and carotid artery stiffness [99]. Fujimoto and his coworkers found that one year of endurance training failed to improve the cardiac output [100]. These studies suggested that ET plays an important role in the improvement of exercise capacity and QoL in the HFPEF population. The mechanisms are probably associated with increased function of metabolically active skeletal muscles instead of cardiac factors [101]. Other nonpharmacological treatments are focused on device therapies. The RESET study aimed to assess the potential benefit of rate-adaptive pacing (RAP) in patients with mild-to-moderate HFPEF based on the prevalence of chronotropic incompetence in HFPEF but failed to enroll sufficient patients [102]. Moreover, the increase in parasympathetic tone has been suggested as a treatment for autonomic dysfunction in patients with HFPEF by carotid sinus stimulation [103]. The clinical safety and efficacy of chronic baroreflex therapy in patients with HFPEF is being evaluated in the CVRx Health Outcomes Prospective Evaluation for Heart Failure with EF ≥ 40% (HOPE4HF) trial [104]. In addition, some patients with severe HFPEF may be associated with interatrial dyssynchrony. Left atrial (LA) pacing therapy might have beneficial effects on restoration of LV active filling and decrease of LA pressure. This will be assessed in a randomized, controlled crossover “LEAD” study recently [105]. Finally, cardiac resynchronization therapy has been developed since dyssynchrony is common in HFPEF. But this still needs further powerful evidence [106, 107].

## 6. Conclusions

Compared with HFREF, HFPEF is associated with a similar prevalence and mortality, yet no effective treatment has been achieved in RCTs. This may be attributed to unrevealed pathophysiological mechanisms, multiple comorbidities existence, and high noncardiac deaths. Although treatment options remain unclear concerning mortality in patients with HFPEF, most of these patients have significant co-morbidities which are strongly associated with mortality, as well as these comorbidities including hypertension, CAD, diabetes, and CKD. Importantly, these comorbidities have to be treated effectively under the guidance of evidence-based medicine. Therefore, we should identify and treat these comorbidities positively instead of waiting for the new findings of treatments in HFPEF [108]. Likewise, while regarding the goal of treatment, HFPEF patients are often older, and improvements of endpoints such as functional exercise capacity and QoL may be more important than mortality only. Fortunately, putting aside mortality, current results of clinical trials in patients with HFPEF have shown positive evidence of exercise capacity improvement [47]. But despite all this, new pathophysiological concepts and improved diagnostic algorithms are still needed to generate new therapeutic options in the future. Finally, it is necessary to continue further ongoing studies that could be helpful to increase our understanding of the pathophysiology and develop novel therapeutic strategies in the HFPEF population.
